# Identification of Binding Sites in Huntingtin for the Huntingtin Interacting Proteins HIP14 and HIP14L

**DOI:** 10.1371/journal.pone.0090669

**Published:** 2014-02-28

**Authors:** Shaun S. Sanders, Katherine K. N. Mui, Liza M. Sutton, Michael R. Hayden

**Affiliations:** Department of Medical Genetics and Centre for Molecular Medicine and Therapeutics, Child and Family Research Institute, University of British Columbia, Vancouver, British Columbia, Canada; Hokkaido University, Japan

## Abstract

Huntington disease is an adult onset neurodegenerative disease characterized by motor, cognitive, and psychiatric dysfunction, caused by a CAG expansion in the *HTT* gene. Huntingtin Interacting Protein 14 (HIP14) and Huntingtin Interacting Protein 14-like (HIP14L) are palmitoyl acyltransferases (PATs), enzymes that mediate the post-translational addition of long chain fatty acids to proteins in a process called palmitoylation. HIP14 and HIP14L interact with and palmitoylate HTT and are unique among PATs as they are the only two that have an ankyrin repeat domain, which mediates the interaction between HIP14 and HTT. These enzymes show reduced interaction with and palmitoylation of mutant HTT, leading to increased mutant HTT inclusion formation and toxicity. The interaction between HIP14 and HTT goes beyond that of only an enzyme–substrate interaction as HTT is essential for the full enzymatic activity of HIP14. It is important to further understand and characterize the interactions of HTT with HIP14 and HIP14L to guide future efforts to target and enhance this interaction and increase enzyme activity to remediate palmitoylation of HTT and their substrates, as well as to understand the relationship between the three proteins. HIP14 and HIP14L have been previously shown to interact with HTT amino acids 1–548. Here the interaction of HIP14 and HIP14L with N- and C-terminal HTT 1–548 deletion mutations was assessed. We show that HTT amino acids 1–548 were sufficient for full interaction of HTT with HIP14 and HIP14L, but partial interaction was also possible with HTT 1–427 and HTT 224–548. To further characterize the binding domain we assessed the interaction of HIP14-GFP and HIP14L-GFP with 15Q HTT 1-548Δ257-315. Both enzymes showed reduced but not abolished interaction with 15Q HTT 1-548Δ257-315. This suggests that two potential binding domains exist, one around residues 224 and the other around 427, for the PAT enzymes HIP14 and HIP14L.

## Introduction

Huntington disease (HD) is an autosomal dominant neurodegenerative disease characterized by motor, cognitive, and psychiatric dysfunction with onset in mid-life and death following, on average, 20 years later [1], [2]. The striatum is the brain region to first undergo neurodegeneration with more widespread pathology occurring at later stages of the disease [1], [2]. HD is caused by a CAG expansion in exon 1 of the *HTT* gene that results in a poly-Q expansion in the HTT protein (NP_002102) [3].

One approach that has been taken to determine the functions of the HTT protein and to understand the pathogenesis of HD is to identify and characterize HTT interacting proteins and to determine how these interactions are altered in the presence of the HD mutation. Huntingtin Interacting Protein 14 (HIP14; ZDHHC17; NP_056151) was first identified as a HTT interactor in a yeast 2-hybrid screen. HIP14 was further shown to interact with HTT in mammalian systems and to interact less with mutant HTT (mHTT) [4], [5]. The HIP14 homolog Huntingtin Interacting Protein 14-like (HIP14L; ZDHHC13; NP_061901) was first identified based on its high amino acid sequence similarity to HIP14 and was later shown to also be a *bona fide* HTT interactor that also interacts less with mHTT [5], [6].

**Figure 1:**
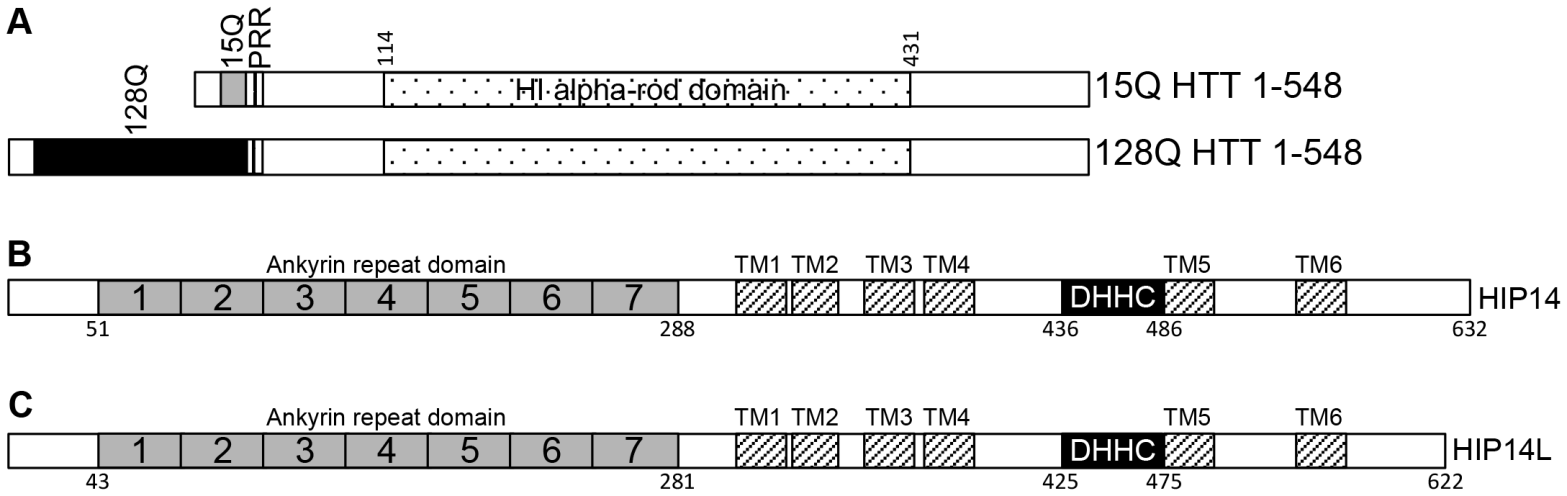
Overview schematics of the domain organization of HTT (A), HIP14 (B), and HIP14L (C). The domain organization of HTT is shown in (**A**) with the poly-glutamine domains of WT (15Q) and mutant (128Q) HTT (NP_002102) are shown in grey and black rectangles, respectively, the proline rich repeat is shown in a hatched rectangle, and the H1 alpha-rod domain is shown in a dotted rectangle with the amino acids indicated above. (**B**) The domain organization of HIP14 (NP_056151) is shown in (**B**) and of HIP14L (NP_061901) in (**C**) with the 7 ankyrin repeats making up the ankyrin repeat domain shown in numbered solid grey rectangles, the transmembrane domains shown in hatched rectangles labeled TM1-6, and the DHHC cysteine-rich domain shown in solid black rectangles labeled DHHC. The amino acids corresponding to the appropriate domains are indicated below.

HIP14 and HIP14L both belong to the 23 member family of DHHC (Asp-His-His-Cys) cysteine-rich (DHHC-CR) domain-containing palmitoyl acyltransferases (PATs) [7], [8]. DHHC-CR PATs are a family of enzymes that mediate post-translational S-acylation of proteins, involving the addition of long chain fatty acids to proteins at cysteine residues via a thioester bond. S-acylation is commonly referred to as palmitoylation because palmitate is the most common long chain fatty acid in the cell [9], [10]. Many proteins, including HTT, are dynamically palmitoylated and palmitoylation modulates membrane localization, function, protein-protein interactions, and other post-translational modifications of palmitoyl-proteins [11]. HIP14 and HIP14L are unique among the DHHC-CR PATs as they are the only two that have six transmembrane domains (TMDs) and seven ankyrin repeats (Figure 1B and C). The ankyrin repeat domain is believed to mediate the interaction between HIP14 and HTT [11], [12].

HIP14 and HIP14L are not only HTT interactors but they are also the primary PATs for HTT [12]. These PATs show reduced interaction and palmitoylation of mHTT leading to increased mHTT inclusion formation and toxicity [13]. Interestingly, both the *Hip14-* and *Hip14l-*deficient mouse models recapitulate many HD-like phenotypes suggesting that both proteins may play a role in the pathogenesis of HD [6], [14]. Indeed, HIP14 has been shown to be dysfunctional in the presence of the HD mutation or upon loss of wild type HTT, making it unable to effectively palmitoylate its substrates SNAP25 and GluR1 [12], [14]. These data suggest that the interaction between HIP14 and HTT goes beyond that of only a enzyme-substrate interaction and that HTT is essential for the full enzymatic activity of HIP14 [12], [14]. HIP14L is structurally very similar to HIP14, containing all the same domains in the same orientation; thus it is possible that HTT also modulates the function of HIP14L.

It is important to further understand and characterize the interactions of HTT with HIP14 and HIP14L to guide future efforts to target and enhance this interaction to increase enzyme activity and remediate palmitoylation of HTT and their substrates. It is important to know if HIP14 and HIP14L interact with the same domain of HTT and, if so, if they compete for binding. A shared binding site would provide further support for the hypothesis that these two PATs are able to compensate for each other in palmitoylating HTT and that HTT may also modulate the activity of HIP14L. If they were to compete for binding, this would need to be taken into consideration when taking efforts to increase the interaction between HTT and one PAT or the other at the risk of decreasing the interaction with the other PAT. HIP14 has been previously shown to interact with HTT amino acids 1–548 (HTT 1–548) [12]. Here, amino (N)- and carboxy (C)-terminal deletions of HTT 1–548 were generated and their interaction with HIP14 and HIP14L was assessed to determine the location of the binding site.

## Materials and Methods

### Plasmids and Cloning

The generation of HIP14-GFP (NM_015336) and HIP14L-GFP (NM_001001483), 15Q and 128Q HTT 1–548 (15Q and 128Q 1955; NM_002111), and HTT 1–427 (1597) and HTT 1–224 (989) was described previously [6], [7], [15], [16]. The C-terminal deletion mutants were generated by PCR cloning using the indicated primers in Table 1. The primers (Integrated DNA technologies) had EcoRI and NotI restriction enzyme sites added on the 5′ and 3′ sides of the PCR product respectively and the forward primers also had a start codon added. This allowed the PCR products to be digested and ligated into the EcoRI and NotI sites of pCI-neo (enzymes from New England Biolabs; pCI-neo from Promega). 15Q HTT 1-548Δ257-315 was generated by insertion of a *HTT* gBlock gene fragment into the BlpI and Bsu36I restriction enzyme sites following digestion with the same enzymes such that amino acids 257–315 were deleted. All clones were confirmed by sequencing.

**Table 1:** Cloning primers used to generate the HTT 1–548 N-terminal deletion mutants.

Primer name	Sequence
HTT N-term reverse	tcccatctgaccctgccatg **tga** *gcggccgc*tactgctatg
HTT 427–548 forward	tcgtacttat*gaattc* **atg** ggagggggttcctcatgcag
HTT 224–548 forward	tcgtacttatgaattc**atg** tcagtccaggagaccttggc
HTT 151–548 forward	tcgtacttat*gaattc* **atg** tgcctcaacaaagttatcaa
HTT 88–548 forward	tcgtacttat*gaattc* **atg** cgaccaaagaaagaactttc

*Restriction enzyme sites are in italics (EcoRI in forward primers and NotI in the reverse), the start and stop codons are in bold, and the primer binding sequence is underlined.

### Antibodies

The primary antibodies used were GFP goat polyclonal antibody (sc-5385, Santa Cruz Biotechnology, 1∶50 for immunoprecipitation), HTT mouse monoclonal antibody (MAB2166, Millipore, 1∶1000 for immunoblotting), HTT mouse monoclonal antibody (in-house BKP1, 1∶100 for immunoblotting), and GFP rabbit polyclonal antibody (EU2, Eusera, 1∶10000 for immunoblotting). Fluorescently conjugated secondary antibodies for immunoblotting used were Alexa Fluor 680 goat anti-Rabbit (A21076, Molecular Probes, 1∶10000) and IRDye 800CW goat anti-Mouse (610-131-121, Rockland, 1∶2500).

### Cell Culture and Transfection

Cells were cultured in DMEM with 10% fetal bovine serum, penicillin/streptomycin (1000 Units/mL Penicillin and 1000 ug/mL streptomycin), and 2 mM L-glutamine at 37°C in 5% CO_2_ (Gibco). Constructs were transiently transfected in COS-7 cells (ATCC) with X-tremeGENE 9 DNA transfection reagent (Roche) according to the manufacturer’s instructions. Cells were harvested after 24 h for co-immunoprecipitation experiments described below.

### Cell Lysis and Co-immunoprecipitations

Cells were homogenized on ice for 5 min in one volume 1% SDS TEEN [TEEN: 1 M Tris pH 7.5, 0.5 M EDTA, 0.5 M EGTA, 3 M NaCl, 1 mM Complete protease inhibitor cocktail (Roche), 1 mM sodium vanadate, 1 mM phenylmethylsulfonyl fluoride and 5 µM zVAD-FMK] and subsequently diluted in four volumes 1% TritonX-100 TEEN for 5 min for further homogenization. Samples were sonicated at one time at 20% power for 5 seconds to shear DNA and the insoluble material was removed by centrifugation at 14000 revolutions per minute for 15 min. Samples were immunoprecipitated overnight with Dynabeads Protein G (Invitrogen) and antibody.

### Western Blotting Analysis

Proteins in both the cell lysates and immunoprecipitates were heated at 70°C in 1×NuPAGE LDS sample buffer (Invitrogen) with 10 mM DTT before separation by SDS-PAGE. After transfer of the proteins onto nitrocellulose membrane, immunoblots were blocked in 5% milk TBS (TBS: 50 mM Tris pH 7.5, 150 mM NaCl). Primary antibody dilutions of HTT mouse monoclonal antibody and GFP rabbit polyclonal antibody in 5%BSA PBST (Bovine Serum Albumin, Phosphate Buffered Saline with 5% Tween-20) were applied to the immunoblots at 4°C overnight. Corresponding secondary antibodies were applied in 5% BSA PBST for an hour. Fluorescence was scanned and quantified with Odyssey Infrared Imaging system (Li-COR Bioscience) and quantified using the Li-COR software. All error bars are standard error of mean.

## Results

### Deletion of HTT Amino Acids 224–548 Abolishes the Interaction of HTT with HIP14 and HIP14L

**Figure 2:**
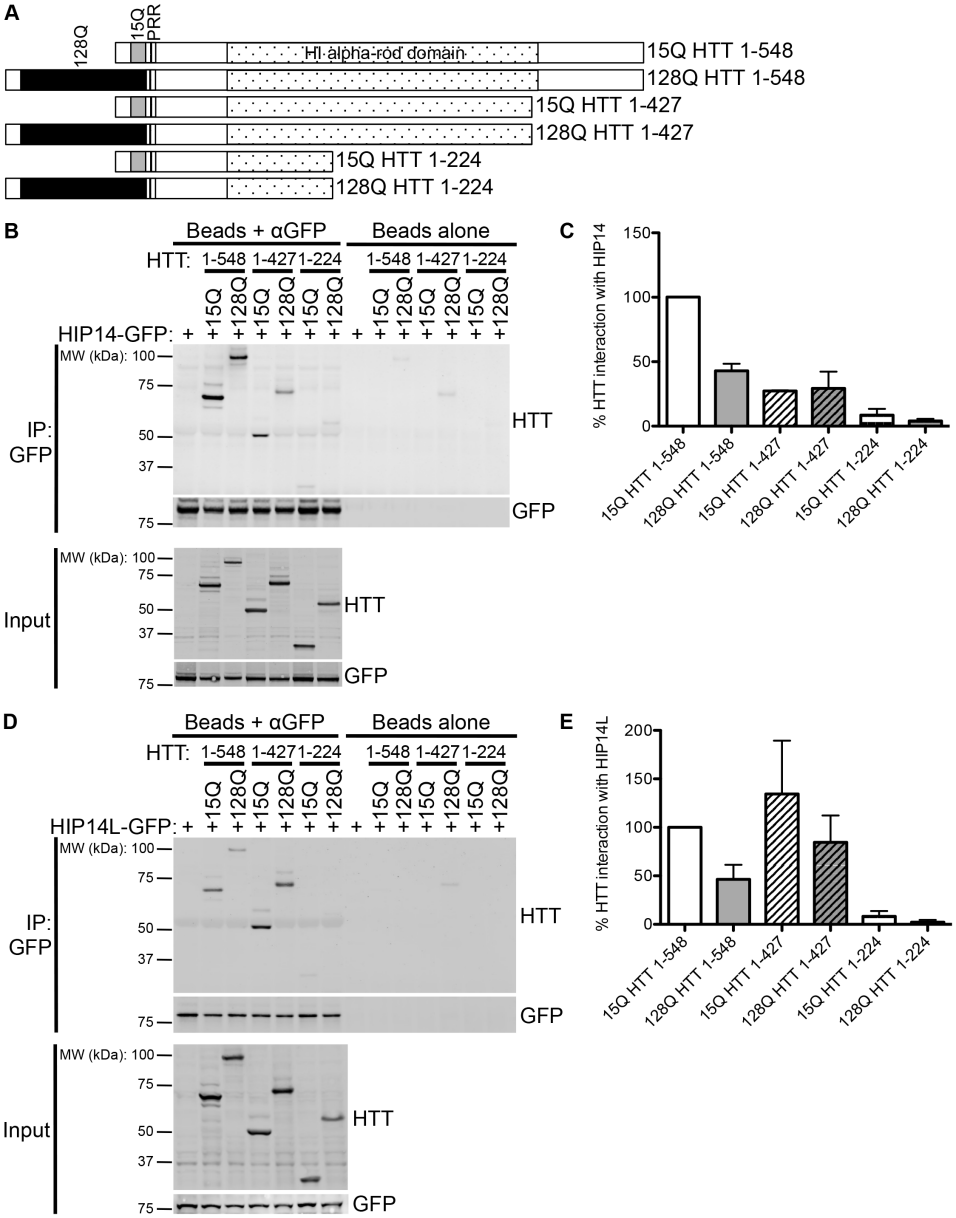
HIP14 and HIP14L interaction with C-terminal deletion mutants of HTT 1-548. (**A**) A schematic diagram of the HTT 1–548 C-terminal deletion mutants used in co-immunoprecipitation experiments with HIP14-GFP and HIP14L-GFP showing the 15Q or 128Q poly-Q domains, the proline rich region (PRR), and the H1 alpha-rod domain. (**B**) A representative image (top two panels) of the co-immunoprecipitation between these C-terminal deletion mutants and HIP14-GFP where GFP was immunoprecipitated and the resulting blots were probed for HTT (top panel) and GFP (bottom panel) showing less 15Q and 128Q HTT 1–427 co-immunoprecipitated with HIP14-GFP. On the right is a beads alone (no antibody) control showing no non-specific binding of the proteins to the beads. The bottom two images show the expression of the HTT deletion mutants (top panel) and of HIP14-GFP (bottom panel). (**C**) Quantification of three independent co-immunoprecipitation experiments where the % HTT interaction with HIP14 is the indicated HTT band intensity as a percentage of the HIP14-GFP band intensity from the same sample, normalized to 15Q HTT 1–548. (**D**) A representative image (top two panels) of the co-immunoprecipitation between the HTT 1–548 C-terminal deletion mutants and HIP14L-GFP where GFP was immunoprecipitated and the resulting blots were probed for HTT (top panel) and GFP (bottom panel). Less 15Q and 128Q HTT 1–427 co-immunoprecipitated with HIP14L-GFP. On the right is a beads alone (no antibody) control showing no non-specific binding of the proteins to the beads. The bottom two panels show the expression of the HTT deletion mutants (top panel) and of HIP14L-GFP (bottom panel). (**E**) Quantification of three independent co-immunoprecipitation experiments where the % HTT interaction with HIP14L-GFP is the indicated HTT band intensity as a percentage of the HIP14L-GFP band intensity from the same sample, normalized to 15Q HTT 1–548.

HIP14 was previously shown to interact with HTT 1–548 [5]. The domain organization of HTT 1–548 15Q and 128Q is shown in Figure 1A with the poly-Q, the proline rich region, and the H1 alpha-rod domain indicated [17]. HIP14 was previously shown in a yeast 2-hybrid experiment to have reduced interaction with HTT 1–427 compared to its interaction with HTT 1–548 and no interaction with HTT 1–224, HTT 1–151, HTT 1–88, and HTT 1–40 [12]. However, as this interaction analysis was performed in yeast it was repeated here in a mammalian system using the mammalian expression versions of the constructs used in the yeast 2-hybrid experiments [12], [16]. Conveniently, these truncation spots remove the C-terminal region upstream of the H1 alpha-rod domain (HTT 1–427) or this C-terminal region and half of the H1 alpha-rod domain (HTT 1–224) (Figure 2A). HTT 1–548 and two C-terminal deletion mutants, 15Q or 128Q HTT 1–427 and 15Q or 128Q HTT 1–224 (Figure 2A), were transiently co-expressed with HIP14-GFP or HIP14L-GFP expressing constructs in COS-7 cells. GFP was immunoprecipitated and resulting blots were probed with antibodies to detect GFP and HTT. As previously shown, 57% less mutant HTT 1–548 (128Q HTT 1–548) co-immunoprecipitated with HIP14-GFP than WT HTT 1–548 (15Q HTT 1–548), indicating reduced interaction in the presence of the HD mutation (Figure 2B and C; n = 3). Both 15Q and 128Q HTT 1–427 exhibited decreased, but not abolished, interaction with HIP14-GFP while both 15Q and 128Q HTT 1–224 interacted very little or not at all with HIP14-GFP (Figure 2B and C; n = 3). The same interaction pattern was observed with a HIP14-FLAG tagged construct and HTT 1–548 did not interact with GFP alone (data not shown). All further experiments were performed using the GFP tagged constructs as they express better in COS cells. These data indicate that HTT 1-427 is required for full interaction with HIP14 and the interaction is abolished with deletion of amino acids 224–548 in the HTT 1–224 truncation protein. No interaction between HIP14-GFP and HTT 1–151, HTT 1–88, or HTT 1–40 was observed (data not shown).

As the domain of HTT that interacts with HIP14L has never been determined, the same co-immunoprecipitation experiment between HIP14L-GFP and the above-mentioned HTT C-terminal deletion mutants, 15Q or 128Q HTT 1–427 and 15Q or 128Q HTT 1–224 was performed. Similar to the results obtained with HIP14, 128Q HTT 1–548 interacted much less with HIP14L-GFP than did 15Q HTT 1–548, indicating reduced interaction with mutant HTT (54% decrease; Figure 2D and E; n = 3). However, no change in interaction of HIP14L with the 15Q and 128Q HTT 1–427 deletion mutants was observed in co-immunoprecipitation experiments (Figure 2D and E; n = 4). The HTT 1–224 deletion mutant did not interact with HIP14L (Figure 2D and E; n = 3). No interaction between HIP14L-GFP and HTT 1–151, HTT 1–88, or HTT 1–40 was observed (data not shown). These data indicate that HTT 1–427 is sufficient for interaction with HIP14L and the interaction is abolished with deletion of amino acids 224–548 in the HTT 1–224 truncation protein.

### Deletion of HTT Amino Acids 1–427 Abolishes the Interaction of HTT with HIP14 and HIP14L

**Figure 3:**
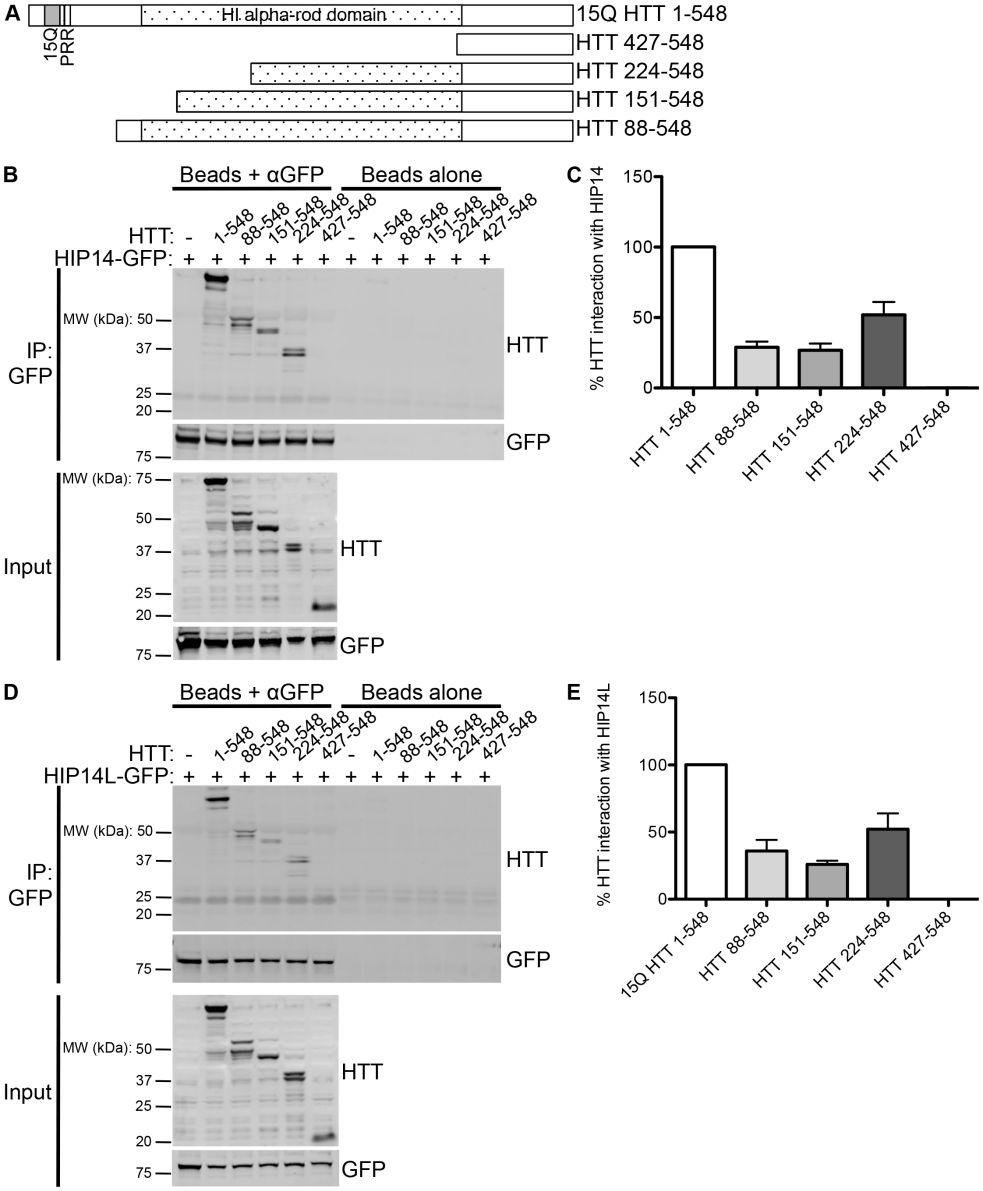
HIP14 and HIP14L interaction with N-terminal deletion mutants of HTT 1–548. (**A**) A diagram of the HTT 1–548 N-terminal deletion mutants used in co-immunoprecipitation experiments with HIP14-GFP and HIP14L-GFP showing the 15Q poly-Q domains, the proline rich region (PRR), and the H1 alpha-rod domain. (**B**) A representative image (top two panels) of the co-immunoprecipitation between these N-terminal deletion mutants and HIP14-GFP where GFP was immunoprecipitated and the resulting blots were probed for HTT (top panel) and GFP (bottom panel) showing less HTT 88–548, HTT 151–548, and HTT 224–548 co-immunoprecipitated with HIP14-GFP and no HTT 427–548 was co-immunoprecipitated with HIP14. On the right is a beads alone control showing no non-specific binding of the proteins to the beads. The bottom two images show the expression of the HTT deletion mutants (top panel) and of HIP14-GFP (bottom panel). (**C**) Quantification of three co-immunoprecipitation experiments where the % HTT interaction with HIP14 is the indicated HTT band intensity as a percentage of the HIP14-GFP band intensity from the same sample, normalized to 15Q HTT 1–548. (**D**) A representative image (top two panels) of the co-immunoprecipitation between the HTT 1–548 N-terminal deletion mutants and HIP14L-GFP where GFP was immunoprecipitated and the resulting blots were probed for HTT (top panel) and GFP (bottom panel). Less HTT 88–548, HTT 151–548, and HTT 224–548 and no HTT 427–548 was co-immunoprecipitated with HIP14L-GFP. On the right is a beads alone control showing no non-specific binding of the proteins to the beads. The bottom two panels show the expression of the HTT deletion mutants (top panel) and of HIP14L-GFP (bottom panel). (**E**) Quantification of three co-immunoprecipitation experiments where the % HTT interaction with HIP14L-GFP is the indicated HTT band intensity as a percentage of the HIP14L-GFP band intensity from the same sample, normalized to 15Q HTT 1–548.

To further characterize the domain of interaction of HTT with HIP14 or HIP14L, N-terminal deletion mutants complementary to the C-terminal deletion mutants were generated; HTT 88–548, HTT 151–548, HTT 224–548, and HTT 427–548 (Figure 3A). These deletion mutants were transiently co-expressed with HIP14-GFP or HIP14L-GFP in COS-7 cells. Reduced interaction of HTT with HIP14-GFP was observed with HTT 88–548, HTT 151–548, and HTT 224–548 and the interaction was abolished upon the deletion of amino acids 1–427 in the HTT 427–548 deletion mutant (Figure 3B and C; n = 3). These data indicate that HTT amino acids 224 to 548 are sufficient for partial interaction with HIP14.

A similar effect was observed between the interaction of HIP14L and the HTT C-terminal deletion mutants as with HIP14. Reduced interaction of HTT 88–548, HTT 151–548, and HTT 224–548 with HIP14L-GFP was observed in similar co-immunoprecipitation experiments and again complete loss of interaction was observed with the HTT 427–548 deletion mutant (Figure 3D and E; n = 3). These data also indicate that HTT amino acids 224 to 548 are sufficient for partial interaction with HIP14L and, along with the data discussed above, suggests that there may be a HIP14/HIP14L binding domain between amino acids 224–427 of HTT.

### Deletion of Amino Acids 257–315 of HTT does not Abolish the Interaction with HIP14 and HIP14L

**Figure 4:**
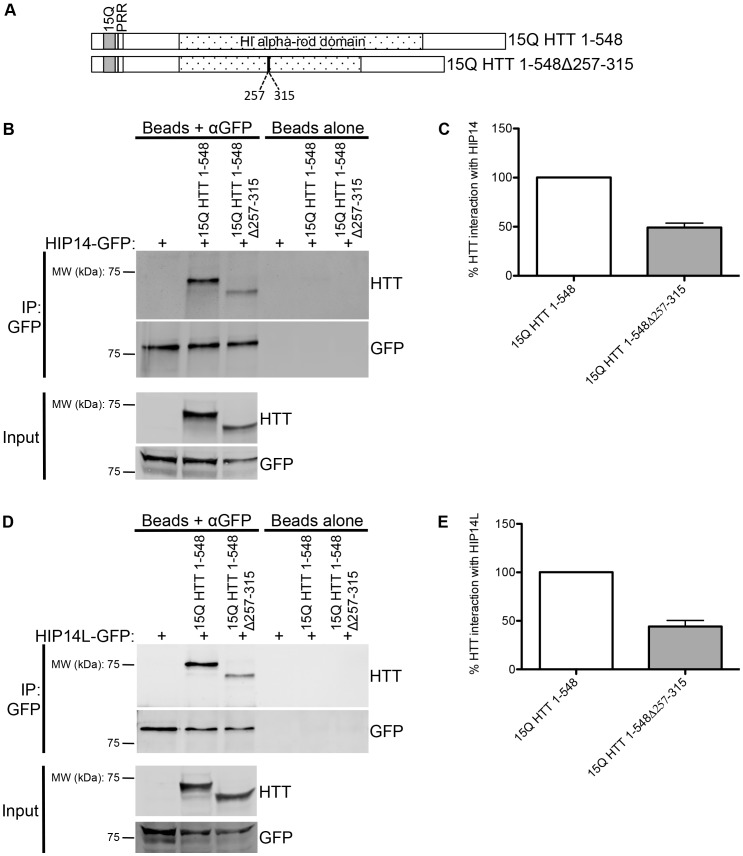
HIP14 and HIP14L interaction with 15Q HTT 1-548Δ257-315. (**A**) A diagram of the 15Q HTT 1-548Δ257-315 deletion mutant used in co-immunoprecipitation experiments with HIP14-GFP and HIP14L-GFP showing the 15Q poly-Q domains, the proline rich region (PRR), and the H1 alpha-rod domain. (**B**) A representative image (top two panels) of the co-immunoprecipitation between 15Q HTT 1-548Δ257-315 deletion mutant and HIP14-GFP where GFP was immunoprecipitated and the resulting blots were probed for HTT (top panel) and GFP (bottom panel) showing less of the 15Q HTT 1-548Δ257-315 deletion mutant co-immunoprecipitated with HIP14-GFP and compared to 15Q HTT 1–548. On the right is a beads alone (no antibody) control showing no non-specific binding of the proteins to the beads. The bottom two images show the expression of the 15Q HTT 1-548Δ257-315 deletion mutant (top panel) and of HIP14-GFP (bottom panel). (**C**) Quantification of three independent co-immunoprecipitation experiments where the % HTT interaction with HIP14 is the indicated HTT band intensity as a percentage of the HIP14-GFP band intensity from the same sample, normalized to 15Q HTT 1–548. (**D**) A representative image (top two panels) of the co-immunoprecipitation between the 15Q HTT 1-548Δ257-315 deletion mutant and HIP14L-GFP where GFP was immunoprecipitated and the resulting blots were probed for HTT (top panel) and GFP (bottom panel). Less 15Q HTT 1-548Δ257-315 deletion mutant was co-immunoprecipitated with HIP14L-GFP. On the right is a beads alone (no antibody) control showing no non-specific binding of the proteins to the beads. The bottom two panels show the expression of the 15Q HTT 1-548Δ257-315 deletion mutant (top panel) and of HIP14L-GFP (bottom panel). (**E**) Quantification of three independent co-immunoprecipitation experiments where the % HTT interaction with HIP14L-GFP is the indicated HTT band intensity as a percentage of the HIP14L-GFP band intensity from the same sample, normalized to 15Q HTT 1–548.

One potential mechanism of binding of HIP14 and HIP14L to HTT within amino acids 224–427 is a putatively methylated lysine at K262 within a LKS motif (in human HTT NP_002102). Gao *et al* determined the crystal structure of the HIP14 ankyrin repeat domain and found that it forms a surface aromatic cage that may bind methylated lysines, much like the ankyrin repeat domains of the G9a and G9a-like protein histone lysine methyltransferases [18]. The K262 of the LKS motif within residues 224–427 of HTT is the only lysine in this region that contains an adjacent serine or threonine like that of the methylated lysine of the histone H3 tail sequence, making it a potential site of methylation [18]. To determine if this is a potential binding domain, a HTT deletion protein with amino acids 257–315 deleted, including the LKS motif, was generated (Figure 4A). This deletion mutant was transiently co-expressed with HIP14-GFP or HIP14L-GFP in COS-7 cells. Reduced but not abolished interaction of 15Q HTT 1-548Δ257-315 with HIP14-GFP and HIP14L-GFP was observed (Figure 4B and C for HIP14 and D and E for HIP14L; n = 3). These data indicate that the HIP14/HIP14L binding domain in HTT is not within these amino acids.

## Discussion

HIP14 and HIP14L are HTT interacting and palmitoylating proteins and their interaction and palmitoylation of HTT are decreased in the presence of mHTT [5], [6], [13]. Interestingly, it appears that the interaction between HIP14 and HTT goes beyond that of only an enzyme-substrate interaction and that HTT actually modulates the enzymatic activity of HIP14 [12], [14]. To further understand and characterize the interactions of HTT with HIP14 and HIP14L it is important to identify the domains of interaction to guide future efforts to target and enhance this interaction to increase enzyme activity and remediate palmitoylation of HTT and their substrates. It is necessary to know if HIP14 and HIP14L interact with the same domain of HTT and, if so, if they compete for binding.

HIP14 was previously shown to interact with HTT 1–548 [12]. Here the interaction between HIP14 and HIP14L with N- and C-terminal HTT 1–548 deletion mutants was characterized. HTT amino acids 1–548 are sufficient for the full interaction of HTT with HIP14 and partial interaction is achieved with amino acids 1–427 and 224–548. Full interaction between HTT and HIP14L was achieved with HTT amino acids 1–548 and 1–427 and partial interaction with 224–427. Amino acids 1–224 or 427–548 of HTT were not sufficient for interaction with HIP14 and HIP14L, indicating that a binding domain is likely to exist between amino acids 224–427. To further characterize this binding region a HTT deletion protein with amino acids 257–315 deleted was generated. Reduced but not abolished interaction of 15Q HTT 1-548Δ257-315 with HIP14-GFP and HIP14L-GFP was observed. These data indicate that the HIP14/HIP14L binding domain in HTT is not within these amino acids but that these amino acids are required for the structural integrity of the actual binding domain.

**Figure 5:**
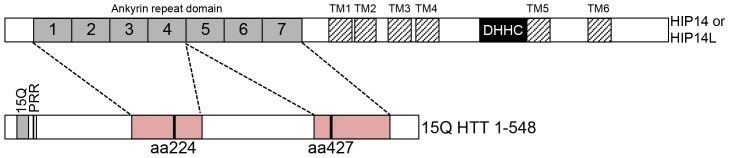
A schematic diagram of the two hypothetical binding scenarios of HTT with HIP14 or HIP14L. In both (**A**) and (**B**) for HIP14 or HIP14L the numbered, solid grey boxes are the seven ankyrin repeats that make up the ankyrin repeat domain, the six TMDs are in hatched boxes labeled TM1–TM6, and the DHHC-CR domain is a black box labeled DHHC. (**A**) In this first scenario, the HIP14 and HIP14L HTT binding site (solid pink box) is between amino acids 224–427 and this binding site interacts with the ankyrin repeat domain of HIP14 or HIP14L. (**B**) In an alternate scenario there are two binding sites (solid pink boxes), one between amino acids 1–427 and the other between amino acids 224–548, that both interact with the ankyrin repeat domain.

A larger region of HTT, amino acids 1–548, is required to achieve full interaction, possibly to achieve correct folding and structural stability of the binding domain or because other sequences outside of this region also contribute to the interaction. The full 1–548 amino acids are required for structural integrity and the correct interaction conformation of HTT likely requires interactions between 1–548 N- and C-terminal parts of HTT to form a compact structure. Based on these data, it is likely that there are actually multiple binding sites, one around amino acid 224 and another around amino acid 427, and that both are required for full interaction and all of the amino acids from 1–548 are required for the structural integrity and confirmation of these binding sites (Figure 5; dashed lines).

Interestingly, in the hypothetical 3D structure of HTT proposed by Palidwor *et al*. HTT has 3 alpha-rod domains (H1-3) that fold back on and interact with each other and interact with HTT interacting proteins (Figure 1A) [17]. The two potential binding domains around residues 224 and 427 are contained within a single structural element of HTT, the H1 alpha-rod domain (resides within amino acids 114–431; Figure 1A). It would be logical that the PAT binding domain would be contained within a single structural element such as the H1 domain thus favoring our model that the PAT binding domains are fully contained within this structural element (Figure 5) [17]. This is consistent with the data presented here where the full 1–548 HTT protein is required for the correct confirmation of this large structural domain and of the two binding sites contained within.

This study identified two potential binding domains around residues 224 and 427 for the PAT enzymes HIP14 and HIP14L. Further characterization of the interactions of HTT with HIP14 and HIP14L is important, as this interaction is believed to go beyond that of a simple enzyme-substrate interaction where HTT actually modulates their function and facilitates palmitoylation of HIP14 substrates.

A common binding domain in HTT for HIP14 and HIP14L along with the fact that HIP14L’s domain structure is virtually identical to HIP14, with all the same domains in the same orientation suggests that HTT may also modulate the enzymatic activity of HIP14L [11]. HTT may modulate the function of these enzymes in several ways. First, HTT is an α–solenoid protein made up of HEAT (Huntingtin, Elongation factor 3, protein phosphatase 2A, TOR1) repeats suitable for its function as a scaffolding protein with many protein-protein interactions [12], [17], [19]–[21]. It is possible that HTT may act as a scaffolding protein to bring substrates into close proximity with HIP14 and HIP14L, acting as an essential linker between PATs and their other substrates. Second, HTT may act as an allosteric activator of HIP14 by affecting the conformational structure of HIP14 thereby allowing substrates to access the DHHC active site [12]. Third, as HTT has been shown to be involved in trafficking of organelles along the cytoskeleton, interacting with multiple motor and motor-associated proteins, HTT may be important for trafficking HIP14 and/or HIP14L to particular subcellular locations allowing it to interact with and palmitoylate its substrates [12], [22].

As the interactions between HTT and HIP14 or HIP14L are reduced in HD and these PATs are implicated in the pathogenesis of HD, understanding the nature of their interactions with HTT may guide future efforts to target and enhance this interaction to increase enzyme activity and remediate palmitoylation of HTT and its substrates. These data indicate that HIP14 and HIP14L share a binding site, providing evidence that these two PATs may compensate for each other in palmitoylating HTT and may compete for binding to HTT and other substrates. This needs to be considered when taking efforts to increase the interaction between HTT and HIP14 at the risk of decreasing the interaction with the other, which may have detrimental effects. If HTT acts as an allosteric activator of HIP14 and HIP14L, binding of a small HTT peptide, including the two binding sites, may enhance HIP14 and HIP14L activity in the disease state, which would likely have a beneficial effect by restoring palmitoylation of HTT and other proteins. This would not be possible without knowing which motifs of HTT bind HIP14 and HIP14L and this study brings us much closer to this goal.

## References

[1] Roos RA (2010) Huntington's disease: a clinical review. Orphanet Journal of Rare Diseases 5: 40 10.1186/1750-1172-5-40 21171977 PMC3022767

[2] Sturrock A, Leavitt BR (2010) The Clinical and Genetic Features of Huntington Disease. Journal of Geriatric Psychiatry and Neurology 23: 243–259 10.1177/0891988710383573 20923757

[3] The Huntington's disease collaborative research group (1993) A novel gene containing a trinucleotide repeat that is expanded and unstable on Huntington's disease chromosomes. Cell 72: 971–983. 8458085 10.1016/0092-8674(93)90585-e

[4] Kalchman MA, Graham RK, Xia G, Koide HB, Hodgson JG, et al (1996) Huntingtin is ubiquitinated and interacts with a specific ubiquitin-conjugating enzyme. J Biol Chem 271: 19385–19394. 8702625 10.1074/jbc.271.32.19385

[5] Singaraja RR, Hadano S, Metzler M, Givan S, Wellington CL, et al (2002) HIP14, a novel ankyrin domain-containing protein, links huntingtin to intracellular trafficking and endocytosis. Human Molecular Genetics 11: 2815–2828. 12393793 10.1093/hmg/11.23.2815

[6] Sutton LM, Sanders SS, Butland SL, Singaraja RR, Franciosi S, et al (2013) Hip14l-deficient mice develop neuropathological and behavioural features of Huntington disease. Human Molecular Genetics 22: 452–465 10.1093/hmg/dds441 23077216

[7] Huang K, Yanai A, Kang R, Arstikaitis P, Singaraja RR, et al (2004) Huntingtin-interacting protein HIP14 is a palmitoyl transferase involved in palmitoylation and trafficking of multiple neuronal proteins. Neuron 44: 977–986 10.1016/j.neuron.2004.11.027 15603740

[8] Ohno Y, Kihara A, Sano T, Igarashi Y (2006) Intracellular localization and tissue-specific distribution of human and yeast DHHC cysteine-rich domain-containing proteins. Biochimica et Biophysica Acta (BBA) - Molecular and Cell Biology of Lipids 1761: 474–483 10.1016/j.bbalip.2006.03.010 16647879

[9] Hallak H, Muszbek L, Laposata M, Belmonte E, Brass LF, et al (1994) Covalent binding of arachidonate to G protein alpha subunits of human platelets. J Biol Chem 269: 4713–4716. 8106438

[10] Smotrys JE, Linder ME (2004) Palmitoylation of Intracellular Signaling Proteins: Regulation and Function. Annu Rev Biochem 73: 559–587 10.1146/annurev.biochem.73.011303.073954 15189153

[11] Young FB, Butland SL, Sanders SS, Sutton LM, Hayden MR (2012) Putting proteins in their place: palmitoylation in Huntington disease and other neuropsychiatric diseases. Progress in Neurobiology 97: 220–238 10.1016/j.pneurobio.2011.11.002 22155432

[12] Huang K, Sanders SS, Kang R, Carroll JB, Sutton L, et al (2011) Wild-type HTT modulates the enzymatic activity of the neuronal palmitoyl transferase HIP14. Human Molecular Genetics 20: 3356–3365 10.1093/hmg/ddr242 21636527 PMC3153302

[13] Yanai A, Huang K, Kang R, Singaraja RR, Arstikaitis P, et al (2006) Palmitoylation of huntingtin by HIP14is essential for its trafficking and function. Nat Neurosci 9: 824–831 10.1038/nn1702 16699508 PMC2279235

[14] Singaraja RR, Huang K, Sanders SS, Milnerwood AJ, Hines R, et al (2011) Altered palmitoylation and neuropathological deficits in mice lacking HIP14. Human Molecular Genetics 20: 3899–3909 10.1093/hmg/ddr308 21775500 PMC3177655

[15] Wellington CL, Ellerby LM, Hackam AS, Margolis RL, Trifiro MA, et al (1998) Caspase cleavage of gene products associated with triplet expansion disorders generates truncated fragments containing the polyglutamine tract. J Biol Chem 273: 9158–9167. 9535906 10.1074/jbc.273.15.9158

[16] Hackam AS, Singaraja R, Wellington CL, Metzler M, McCutcheon K, et al (1998) The influence of huntingtin protein size on nuclear localization and cellular toxicity. The Journal of Cell Biology 141: 1097–1105. 9606203 10.1083/jcb.141.5.1097 PMC2137174

[17] Palidwor GA, Shcherbinin S, Huska MR, Rasko T, Stelzl U, et al (2009) Detection of alpha-rod protein repeats using a neural network and application to huntingtin. PLoS Comput Biol 5: e1000304 10.1371/journal.pcbi.1000304 19282972 PMC2647740

[18] Gao T, Collins RE, Horton JR, Zhang X, Zhang R, et al (2009) The ankyrin repeat domain of Huntingtin interacting protein 14 contains a surface aromatic cage, a potential site for methyl-lysine binding. Proteins 76: 772–777 10.1002/prot.22452 19434754 PMC2733225

[19] Takano H, Gusella JF (2002) The predominantly HEAT-like motif structure of huntingtin and its association and coincident nuclear entry with dorsal, an NF-kB/Rel/dorsal family transcription factor. BMC Neurosci 3: 15. 12379151 10.1186/1471-2202-3-15 PMC137586

[20] Seong IS, Woda JM, Song J-J, Lloret A, Abeyrathne PD, et al (2010) Huntingtin facilitates polycomb repressive complex 2. Human Molecular Genetics 19: 573–583 10.1093/hmg/ddp524 19933700 PMC2807366

[21] Li W, Serpell LC, Carter WJ, Rubinsztein DC, Huntington JA (2006) Expression and characterization of full-length human huntingtin, an elongated HEAT repeat protein. J Biol Chem 281: 15916–15922 10.1074/jbc.M511007200 16595690

[22] Caviston JP, Holzbaur ELF (2009) Huntingtin as an essential integrator of intracellular vesicular trafficking. Trends in Cell Biology 19: 147–155 10.1016/j.tcb.2009.01.005 19269181 PMC2930405

